# Conjugate Heat Transfer Analysis for Cooling of a Conductive Panel by Combined Utilization of Nanoimpinging Jets and Double Rotating Cylinders

**DOI:** 10.3390/nano13030500

**Published:** 2023-01-26

**Authors:** Lioua Kolsi, Fatih Selimefendigil, Hatem Gasmi, Badr M. Alshammari

**Affiliations:** 1 Department of Mechanical Engineering, College of Engineering, University of Ha’il, Ha’il City 81451, Saudi Arabia; 2 Department of Mechanical Engineering, College of Engineering, King Faisal University, Al Ahsa 31982, Saudi Arabia; 3 Department of Mechanical Engineering, Celal Bayar University, 45140 Manisa, Turkey; 4 Department of Civil Engineering, College of Engineering, University of Ha’il, Ha’il City 81451, Saudi Arabia; 5 Laboratoire d’Ingénierie Géotechnique, Ecole Nationale d’Ingénieurs de Tunis, Université de Tunis El Manar, Tunis 1007, Tunisia; 6 Department of Electrical Engineering, College of Engineering, University of Ha’il, Ha’il City 81451, Saudi Arabia

**Keywords:** jet impingement, PV, cooling system, nanoparticle, finite element method, subcooling

## Abstract

In this work, double rotating active cylinders and slot nanojet impingement are considered for the cooling system of a conductive panel. Colder surface temperatures of the cylinders are used, while different rotational speeds are assigned for each of the cylinders. The impacts of cylinder rotational speeds, size and distance between them on the cooling performance are evaluated. The rotational effects and size of the cylinders are found to be very effective on the overall thermal performance. At the highest rotational speeds of the cylinders, the average Nusselt number (Nu) rises by about 30.8%, while the panel temperature drops by about 5.84 ${}^{{}^{\circ}}$C. When increasing the cylinder sizes, temperature drops become 7 ${}^{{}^{\circ}}$C, while they are only 1.75 ${}^{{}^{\circ}}$C when varying the distance between the cylinders. Subcooling and nanofluid utilization contributes positively to the cooling performance, while 1.25 ${}^{{}^{\circ}}$C and 10 ${}^{{}^{\circ}}$C temperature drops are found by varying the subcooled temperature and solid volume fraction. An artificial neural network is used for the estimation of maximum and average panel temperatures when double cylinder parameters are used as the input.

## 1. Introduction

In the thermal management of electronic devices and photovoltaic (PV) panels, temperature control is important. PV thermal efficiency depends upon the cell temperature, while many methods have been offered to reduce its value [1,2,3,4]. The utilization of highly conducive fins, channel cooling and heat pipes are among the popular methods [5,6,7,8,9]. Active methods such as using impinging jets and channel cooling provide effective cooling, as compared with some of the passive methods available for PV thermal management. In the review work of Hasanuzzaman et al. [10], different cooling strategies for PV systems are considered. When active cooling methods have been used, efficiency rises of 22% have been reported, while these amounts are only 15.5% when passive methods are considered. As mentioned in the work of Bahaidarah et al. [11], active methods of PV cooling provide higher heat flux dissipation as compared with some passive methods, such as heat sinks and heat pipes. The jet impinging cooling system is one of the most promising technologies to achieve higher heat transfer (HT) performance in diverse thermal systems, including PVs [12,13,14]. The jet to target surface distance, flow rates, nozzle shape, number of slots and distance between the slots are considered as important design parameters, along with the other operating parameters [15,16,17]. In some recent studies, the effectiveness of using jet impinging cooling for PV systems have been shown [18,19,20].

The effectiveness of channel cooling for PV systems can be increased by using different fluid types. Nanofluids (NFs) have been used in diverse thermal engineering systems, including channel cooling for performance improvements [21,22,23,24,25,26]. Many aspects of NF models in cooling have been considered, and the accurate modeling of NF features has been studied [27,28,29,30,31]. Shape factors of nanoparticles (NPs), loading amount and type of NPs in the base fluid are influential on overall thermal performance. In solar applications, NFs have been shown to provide an effective tool for efficiency improvements [32,33,34,35,36]. In PV–thermal systems (PV-T) systems, both NP type and shape were found to determine the electric and thermal efficiency of the system. In the work of Khanafer and Vafai [37], the effects of using NFs on the performance of different solar systems including solar stills, PV-T and solar collectors have been reviewed. Substantial improvements in efficiency have been reported by using NFs in those systems, while cost and safety have been reported as other challenges.

Additional performance improvement in channel cooling can be achieved by using the insertion of obstacles in the channel. In convective heat transfer (C-HT) applications, stationary, moving or rotating objects can be mounted in-channel to interrupt the boundary layers near the walls and enhance the thermal performance [38,39,40,41]. As rotating cylinders are considered, the rotational speed and size of the cylinders can be used to control the flow and HT features. In convection studies, rotating circular cylinders (RCCs) have been used, while profound impacts on the HT enhancement have been reported [42,43]. Many studies considered the utilization of single RCCs in convection. As many cylinders are considered, the spacing between them and the rotational speeds of each cylinder can be used as control parameters, which contributes to the overall HT performance. In jet impinging systems, the utilization of cylinders has been considered in a few studies [44,45], while rotational effects bring additional improvements in the thermal performance.

In this study, a novel cooling system for thermal management and cooling of a conductive panel is considered. The system consists of double RCCs with jet impingement cooling. Another novel aspect is the utilization of isothermal cold surface temperature of the cylinders with nanofluid to achieve the highest cooling performance. Alumina NPs are used in water, while cylindrical-shaped particles are preferred. As the utilization of solar power in PV modules has the problem of efficiency decrease due to the increased cell temperature, this novel cooling system can be used to increase the effectiveness of PV modules. An artificial neural network (ANN)-based modeling approach is used for estimating the impacts of using RCC parameters on the overall performance of the system. Outcomes are also helpful for the development of cooling systems for the thermal management of electronic devices, batteries and much more.

## 2. Conductive Panel Equipped with Jet Impinging and Active Cylinders Cooling System

**Figure 1:**
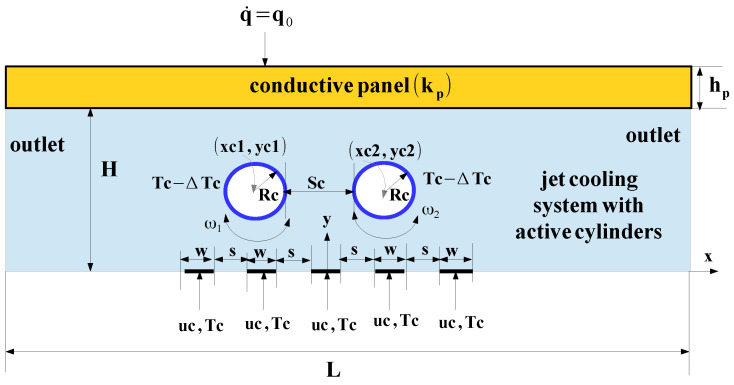
Conductive panel cooling system with slot jet impinging and active RCCs (not to scale).

A jet impinging system with active cylinders is proposed as the cooler of the conductive panel. A schematic view is shown in Figure 1, where double RCCs are considered. Cylinder sizes are Rc, while the distance between them is Sc. Cold fluid enters with uc velocity and Tc temperature. A subcooling is proposed by assigning the temperature of Tc-ΔT for each of the RCCs. Five slots with widths of *w* are used, and the distance between them is s=5w. Here, *H* is the distance between the slots and conducive plate, where $k_{p}$ is the thermal conductivity. Here, *L* and $h_{p}$ denote the length and thickness of the plate. $\dot{q}$ is the uniform heat flux imposed on the plate. The rotational speeds of $\omega_{1}$ and $\omega_{2}$ are considered, while center locations are (xc1,yc1) and (xc2,yc2) for the first and second cylinders. Water–alumina NF is used with cylindrical-shaped NPs, considering various loading amounts up to 3%. We considered a single phase of NF, while impacts of free convection, viscous losses and radiation were ignored. Table 1 shows the NPs and base fluid properties. In the cooling system where RCCs are mounted, the governing equations are given as:

(1) $$\nabla .\left(\rho \vec{V}\right)=0$$

(2) $$\nabla .\left(\rho \vec{V}\vec{V}\right)=-\nabla p+\mu \nabla^{2}\vec{V}$$

(3) $$\nabla .\left(\rho \vec{V}C_{p}T\right)=\nabla .\left(k\nabla T\right).$$

For the panel:

(4) $$\nabla^{2}T=0$$

The jet Reynolds number (Rec), Prandtl number (Pr) and rotational Reynolds number for the first and second cylinders (Rew${}_{1}$, Rew${}_{2}$) and conductivity ratio (KR) are the nondimensional parameters. They are given as:

(5) Re=ρucDhμ,Pr=να,Rew1=ρH(ω1H)μ,Rew2=ρH(ω2H)μ,KR=kpkf

In dimensional form, boundary conditions are given as:

- At the slot inlets: u=uc,T=Tc;
- At the exits: $\frac{\partial u}{\partial x}=\frac{\partial T}{\partial x}=0,v=0$;
- For the first RCC walls:
  u=−ω(y−yc1),v=ω(x−xc1),T=Tc−ΔTc;
- For the second RCC walls:
  u=−ω(y−yc2),v=ω(x−xc2),T=Tc−ΔTc;
- At the cooling system–panel interface:
  ${\left(\frac{\partial T}{\partial n}\right)}_{f}=KR{\left(\frac{\partial T}{\partial n}\right)}_{s},\;\;T_{f}=T_{s}$;
- Jet cooling systems walls: $u=v=0,\;\frac{\partial T}{\partial n}=0$;
- Top part of the panel: $u=v=0,\;\dot{q}=q_{0}$.

The heat flux value is $q_{0}$ = 700 W/m${}^{2}$. For the PV panel, energy balance equations (EBE) are considered. The PV is composed of many layers, such as glass cover, PV cell and back surface. EBE for the glass cover is given as [1]:

(6) $$G\alpha_{g}=h_{rad}\left(T_{g}-T_{s}\right)+h_{v}\left(T_{g}-T_{amb}\right)+k_{T1}\left(T_{g}-T_{c}\right).$$

where glass absorptivity and HT coefficient are given by the terms $\alpha_{g}$, $h_{v}$, while $T_{s}$ is the sky temperature. The EBE for the PV cell is given as [46]:

(7) $$G{\left(\alpha \tau \right)}_{c}+G{\left(\alpha \tau \right)}_{T}\left(1-\beta_{c}\right)=k_{T1}\left(T_{c}-T_{g}\right)+k_{T}\left(T_{c}-T_{bs}\right)+E$$

where the absorptance of the cell and tedlar are given by the terms ${\left(\alpha \tau \right)}_{c}$ and ${\left(\alpha \tau \right)}_{T}$. The packing factor and electric output are given by $\beta_{c}$ and *E*.

The system performance for cooling is given in terms of average Nu (Nu) and average cell temperature ($T_{m}$), which are given as:

(8) $${\mathrm{Nu}}_{s}=\frac{h_{s}D_{h}}{k},\;{\mathrm{Nu}}_{m}=\frac{1}{L}\int_{0}^{L}{\mathrm{Nu}}_{s}ds,\;T_{m}=\frac{1}{A_{s}}\int_{0}^{A_{s}}T_{s}dA,$$

where $h_{s}$ is the local HT coefficient, while *L* and $A_{s}$ are the plate length and surface area of the panel. The local HT is given as:

(9) $$h_{x}=\frac{\dot{q}}{T_{w}-T_{b}}.$$

As for the NF, alumina–water is used, while cylindrical-shaped NPs are considered. The NP solid volume fraction (SVF) is taken up to 3%. Among the important thermophysical properties, the NF thermal conductivity $k_{nf}$ and viscosity $\mu_{nf}$ are given as [47]:

(10) $$\frac{k_{nf}}{\mu_{f}}=1+C_{k}\phi ,$$

(11) $$\frac{\mu_{nf}}{\mu_{f}}=1+A_{1}\phi +A_{2}\phi^{2},$$

where constants $C_{k}$, $A_{1}$ and $A_{2}$ denote the constants for different-shaped NPs. Their values for cylindrical-shaped NPs are given as: $C_{k}=3.95$, $A_{1}=13.5$ and $A_{2}=904.4$.

As the solution method, GWR-FEM (Galerkin weighted residual finite element method) is chosen. In the method, the field variable approximations are made by using:

(12) $$\begin{array}{cc} \; & u=\sum_{k=1}^{N^{u}}\Psi_{k}^{u,v}U_{k},\;v=\sum_{k=1}^{N^{v}}\Psi_{k}^{u,v}V_{k},\\ \; & p=\sum_{k=1}^{N^{p}}\Psi_{k}^{p}P_{k},\;\;T=\sum_{k=1}^{N^{u}}\Psi_{k}^{T}T_{k}, \end{array}$$

where $\Psi^{u,v},\Psi^{p}$ and $\Psi^{T}$ are the shape functions. The related nodal values of the elements are given by the terms U,V,P and *T*. The resulting residual by using a weight function *W* is set to be zero as:

(13) $$\int_{V}WRdv=0$$

by adopting the weight function of *W*. For handling instabilities, the SUPG (Streamline upwind Petrov–Galerkin) method is used, while BICGStab (Biconjugate gradient stabilized) is considered for flow and HT modules of the code. As for the convergence, a value of 10${}^{-7}$ is selected where converged solutions are achieved.

**Figure 2:**
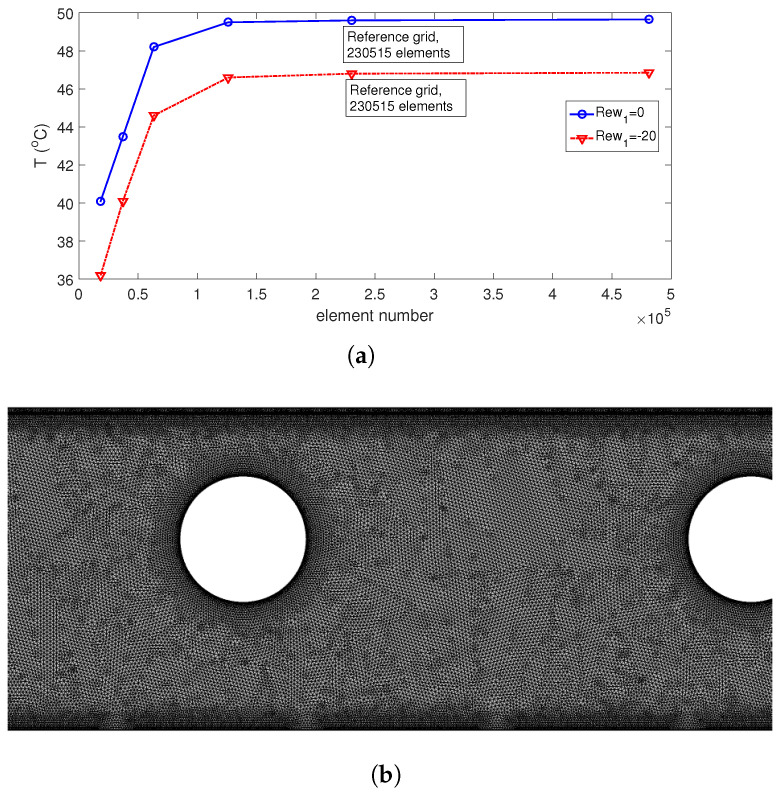
GIT results for average PST at two different rotational speeds of the first cylinder (**a**) and mesh distribution near the RCCs and panel (**b**).

Figure 2a shows the grid independence test (GIT) results, where average surface temperature is considered for different grid sizes at Rew${}_{1}=0$ and Rew${}_{2}=-20$. A grid with 230,515 mixed elements (triangular+quadrilateral) is used. Grid variation near the cylinder and panel is shown in Figure 2b, where near the wall and interface zones, the refinements are considered.

**Figure 3:**
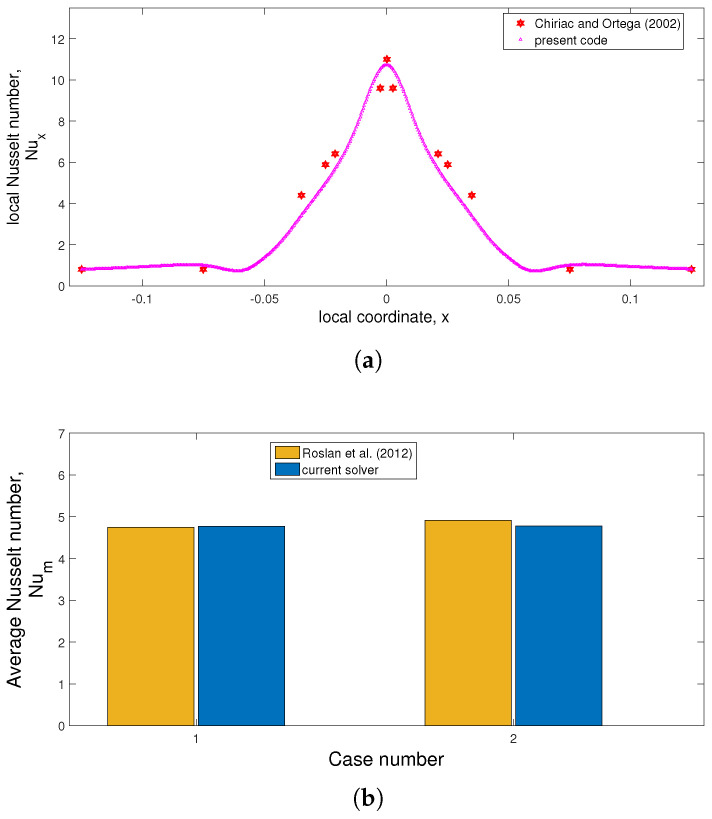
Code validation studies: (**a**) Local Nu distribution comparisons with Ref. [49] at Re = 250 for confined slot jet impingement system and (**b**) average Nu comparisons with Ref. [50] in a differentially heated cavity under the effects of RCC (Case 1 − R=0.1,Ω=500 and Case 2 − R=0.2,Ω=1000).

Validation of the code is made by using different works available in the literature. In the first study, confined slot jet impinging cooling of a hot isothermal surface is considered in the laminar flow regime, as in the work of Chiriac and Ortega [49]. Figure 3a shows the local Nu variation comparisons along the surface at Re = 250. In another work, the impacts of using RCC on the convective HT are examined, as available in the numerical work in ref. [50]. Average Nu variations at two different cylinder sizes and rotational speeds are shown in Figure 3b. The highest deviation below 3% is achieved between the results. These results show that the code can simulate convective HT problems for impinging jets under the effects of RCCs.

## 3. Results and Discussion

This study deals with the application of twin rotating circular cylinders (T-RCC) on the cooling performance of a conductive panel. The outer surfaces of the T-RCCs are isothermal, while lower cooling temperatures can be assigned on both cylinders. Different rotational speeds for the cylinder can be assigned in the range of −20 and 0. The jet Reynolds number (Re) is taken as 300. The conductivity ratio is considered as KR = 250. Temperature difference (dT) between 0 and 10 is considered while both cylinders have the same constant cold temperature. The cooling performance of the panel is explored by varying the size of the cylinders (between 0.01H and 0.2H) and distance between them (between 4Rc and 8Rc). The NP-SVF is taken between 0 and 3%. An artificial neural network (ANN)-based method is used for the development of the input–output relation and performance estimation of the conducive panel under varying RC parameters.

### 3.1. Computational Fluid Dynamics Results

**Figure 4:**
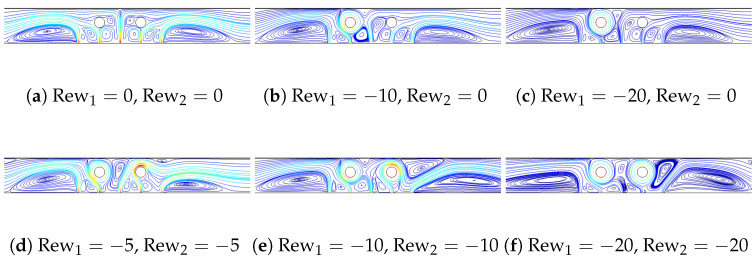
FP variations for different (${\mathrm{Rew}}_{1},{\mathrm{Rew}}_{2}$) combinations at (Rc = 0.1H, Sc = 6Rc, ΔT=10, SVF = 3%).

Figure 4 shows the streamline variations for different combinations of rotational speeds of the first and second cylinder (Rew${}_{1}$, Rew${}_{2}$). When there is no rotation, multiple recirculation zones (RC-Zs) are formed in the vicinity of the cylinders, slots and in between the slots. When rotation of the first cylinder is increased, suppression of the vertices below and above it is seen due to the higher rotational effects. Vortex sizes in between the cylinders and near the right cylinder vary when rotational speeds of twin cylinders are simultaneously increased. In the location where RCCs are closer to the panel, local velocity becomes higher, which results in enhanced thermal transport.

**Figure 5:**
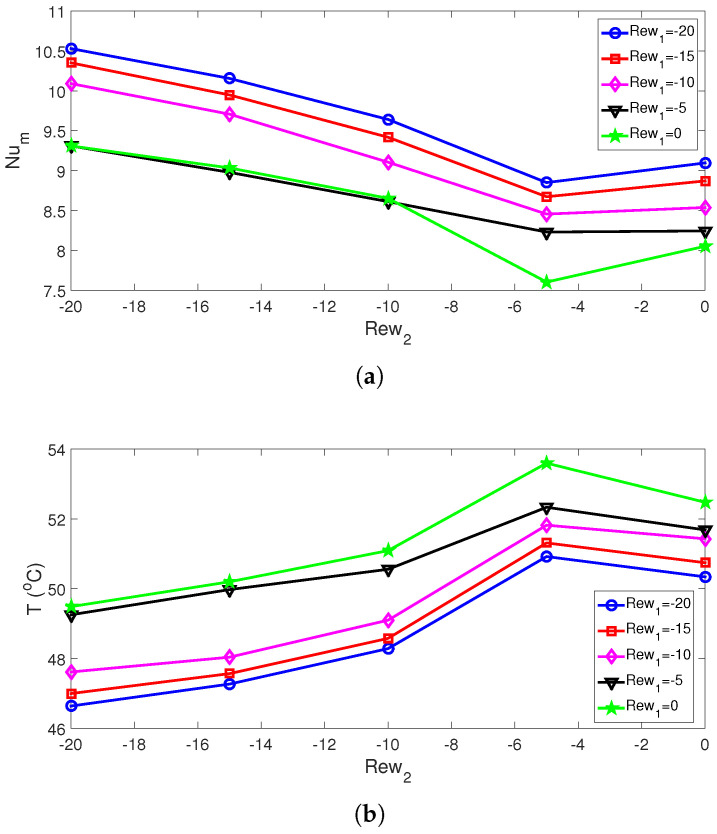
Average Nu (**a**) and average PST (**b**) for different rotational speeds of the cylinders (Rc = 0.1H, Sc = 6Rc, ΔT=10, SVF = 3%).

The cooling performances for the conductive panel are evaluated in terms of average Nu and panel surface temperature (PST). When rotational effects are considered, it generally has a positive impact on the overall cooling performance. In jet cooling systems, the favorable impacts of using RCCs are due to the enhanced thermal transport, due to the rotation of the cylinders. For the Rew${}_{2}$ value between −5 and −20, the average Nu rises for increments in both Rew${}_{1}$ and Rew${}_{2}$. When the stationary reference cases of twin cylinders are considered at (Rew${}_{1}$, Rew${}_{2}$) = (0,0), the average Nu rises by about 13% and 30.8% when rotations are in the cases of twin cylinder rotations (−20,0) and (−20, −20). The resulting reduction in the average PST becomes 2.14 ${}^{{}^{\circ}}$C and 5.84 ${}^{{}^{\circ}}$C (Figure 5).

**Figure 6:**
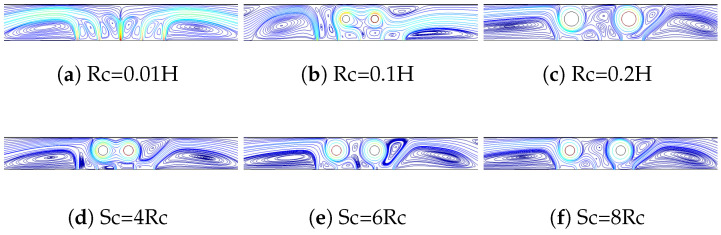
Impacts of RCC size (Sc = 6Rc, (**a**–**c**)) distance between the cylinders (Rc = 0.1H, (**d**–**f**)) on the FP distributions ($\left({\mathrm{Rew}}_{1},{\mathrm{Rew}}_{2}\right)\;=\;\left(-20,-20\right)$, ΔT=10, SVF = 3%.)

Impacts of size and distance between the cylinders on the streamline variations are shown in Figure 6. The rotational effects on the flow pattern become significant when the cylinder size is increased. The vortex size and distribution are significantly influenced by the cylinder size with twin RCCs. At Rc = 0.1H, a vortex is also established near the conductive panel, while its size and location changes with further increment of the cylinder sizes. Near the confined bottom plate, vortex sizes are also varied by the size of the cylinders. When the distance between the cylinders is increased, vertices in between the cylinders are formed, which extend toward the top panel at the highest distance. The vortex near the top of the right cylinder changes its size with higher distances.

**Figure 7:**
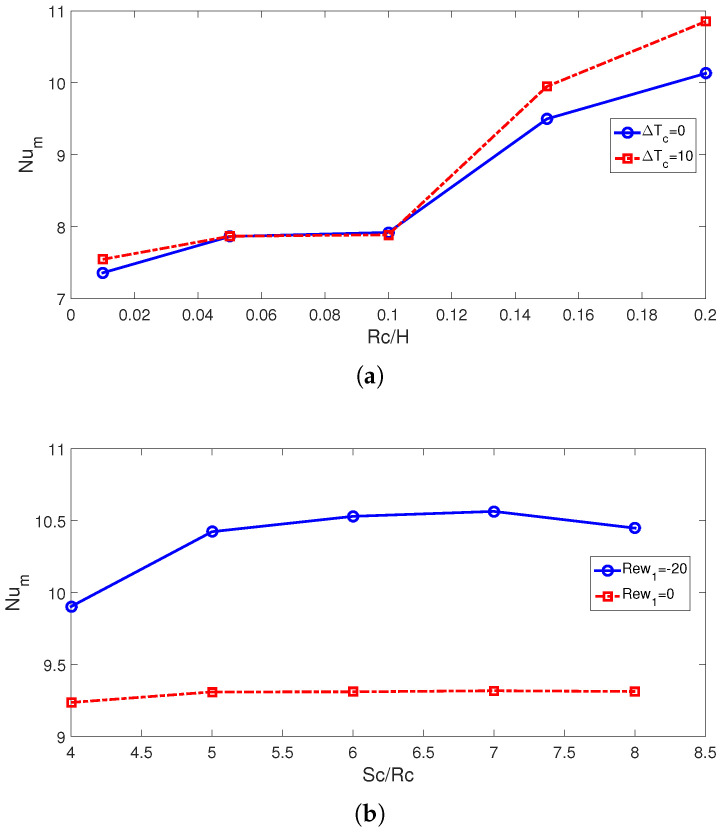
Effects of RCC size (Sc = 6Rc, (**a**)) and distance between the cylinders (Rc = 0.1H, (**b**)) on the average Nu variations ($\left({\mathrm{Rew}}_{1},{\mathrm{Rew}}_{2}\right)\;=\;\left(-20,-20\right)$, ΔT=10, SVF = 3%).

The average Nu rises with higher cylinder sizes. This is attributed to the favorable impacts of rotations when cylinder sizes are larger. The subcooling of the system is achieved by using cylinders which are maintained at lower cold jet temperature of Tc-dTc (Figure 7). The subcooling effects become significant with higher sizes as rotational effects become more pronounced. This is attributed to the positive impact of using a cold rotating surface for which the inlet fluid has a higher temperature to interact with. This impact is effective for larger cylinder size and for higher rotational speeds. Average Nu rises by about 37.8% and 43.9% when the highest RCC size is used at ΔT=0 and ΔT=10. The corresponding average PST drops are evaluated as 6.3 ${}^{{}^{\circ}}$C and 7 ${}^{{}^{\circ}}$C. When no cylinder is used, the average cell temperature is 0.8 ${}^{{}^{\circ}}$C higher as compared with the case with the cylinder having a size of Rc = 0.001H. When rotations are inactive (Rew = 0), the distance between the cylinder has slight impacts on the average Nu, while there is only a 6.7% increase in Nu by varying the distance when RCCs are rotating at Rew = −20. At this rotational speed, the drop in the average PSC becomes 1.75 ${}^{{}^{\circ}}$C when varying the distance between the twin cylinders.

**Figure 8:**
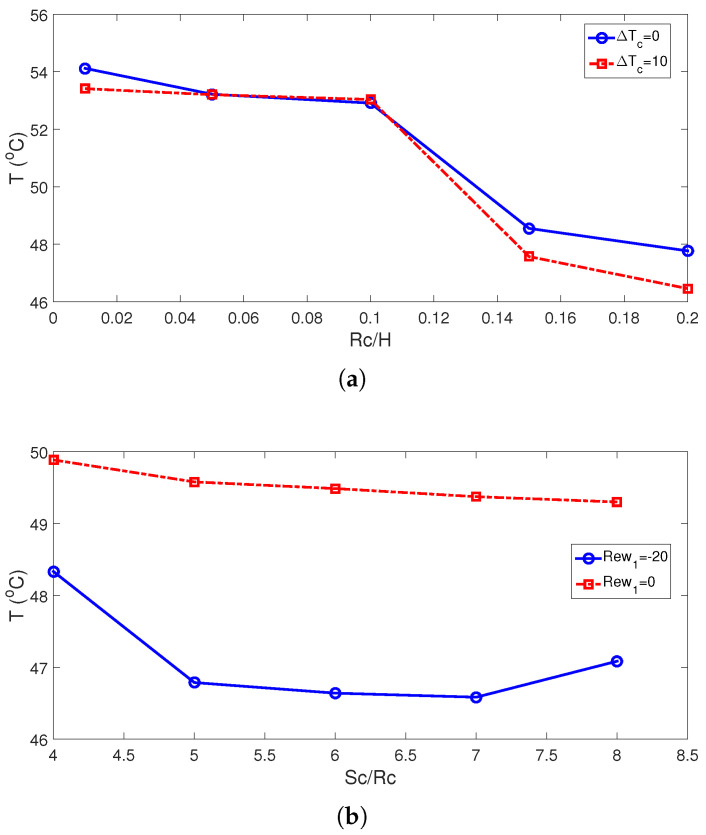
Average PST variations for different RCC sizes (Sc = 6Rc, (**a**)) and for various distances between the cylinders (Rc = 0.1H, (**b**)) ($\left({\mathrm{Rew}}_{1},{\mathrm{Rew}}_{2}\right)\;=\;\left(-20,-20\right)$, ΔT=10, SVF = 3%).

**Figure 9:**
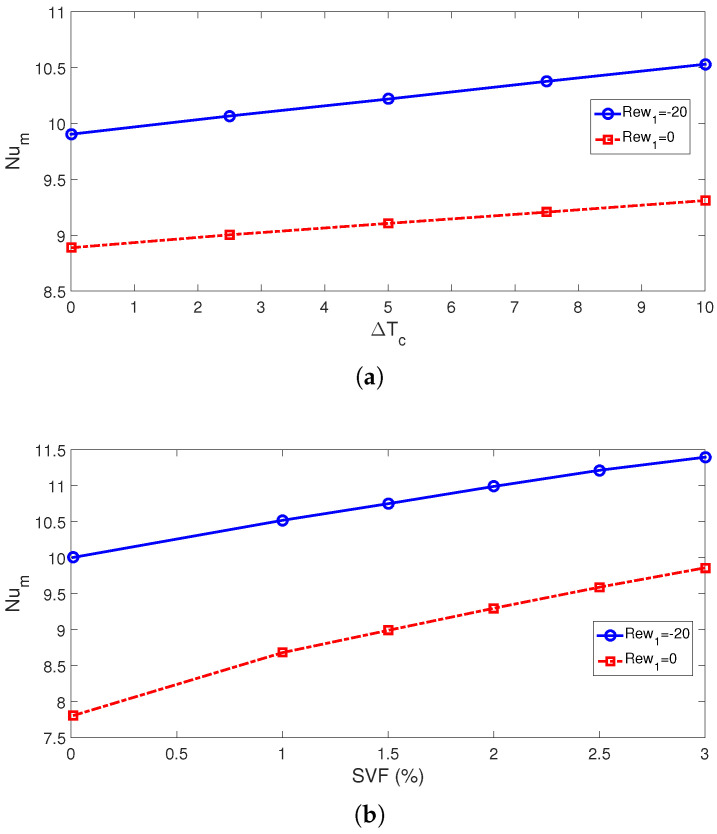
Impacts of subcooled temperature ((**a**), SVF = 3%) and SVF of NPs (ΔT=10, (**b**)) on the variation of average Nu ($\left({\mathrm{Rew}}_{1},{\mathrm{Rew}}_{2}\right)\;=\;\left(-20,-20\right)$, Rc = 0.1H, Sc = 6Rc).

**Figure 10:**
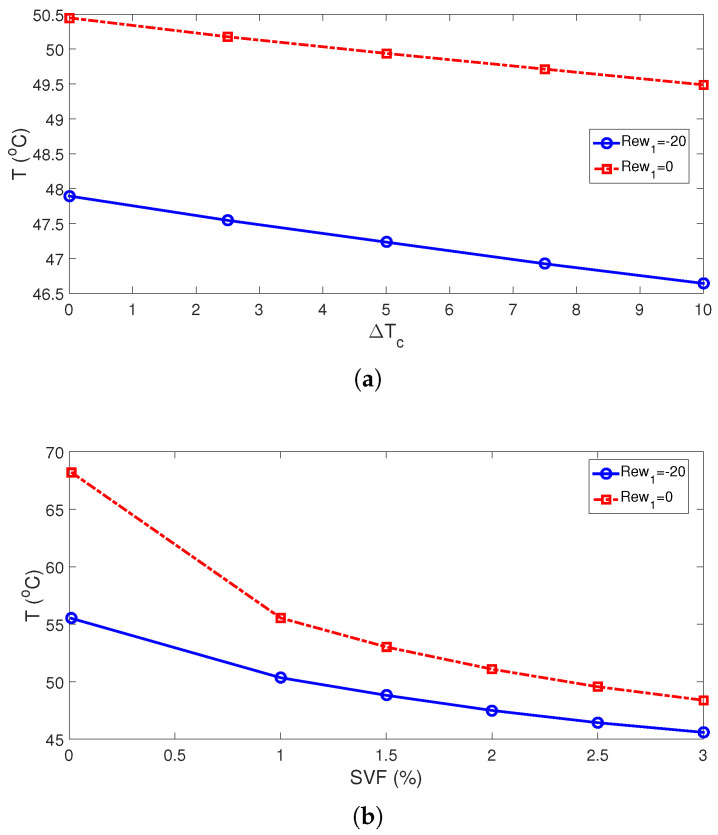
Average panel temperature for varying subcooled temperatures of RCCs ((**a**), SVF = 3%) and SVF of NPs (ΔT=10, (**b**)) ($\left({\mathrm{Rew}}_{1},{\mathrm{Rew}}_{2}\right)\;=\;\left(-20,-20\right)$, Rc = 0.1H, Sc = 6Rc).

Using cooler surface temperatures of the twin cylinders contributed positively to the cooling performance while additional subcooling of the jet system is provided. This is valid both for stationary and rotating cylinder cases, as shown in Figure 8 and Figure 9. There are 5.8% and 6% rises in average Nu by using the highest ΔT for stationary and rotating cylinder configurations, while average PST drops become 1 ${}^{{}^{\circ}}$C and 1.25 ${}^{{}^{\circ}}$C. The cooling performance is further increased by using NF (Figure 9b). Convective HT enhancement with NF and rotating cylinders has been shown in many studies [50,51]. In the present work, water–alumina NF having cylindrical-shaped NPs is used, and different loading amounts in the base fluid are considered. The potential of using different-shaped alumina NPs in base fluid on the HT improvements has been shown in different studies. There is a 26.4% increment in the average Nu for the highest NP-SVF at Rew = 0, while it is 14% at Rew = −20. the highest temperature drop is seen as the NP-SVF rises to 3% when rotations are inactive and, in this case, the average PST drop is 19.8 ${}^{{}^{\circ}}$C. When rotations are used at the highest speed (Rew = −20), the PST value is 10 ${}^{{}^{\circ}}$C (Figure 10).

### 3.2. ANN-Based Performance Estimation

The impacts of using RCCs on the performance of the PV system are estimated by using an ANN-based method. The basics and foundations of the method are very well-established, while many different applications of ANNs in predicting the thermal performance of energy systems have been performed [52,53,54,55,56]. In the preset work, a 4-input/2-output system is considered, where the inputs are the Rew${}_{1}$, Rew${}_{2}$, Rc and Sc, while the outputs are the average and maximum PST. The range of the input and output parameters are given as: $-20\leq {\mathrm{Rew}}_{1}\leq 0,\;-20\leq {\mathrm{Rew}}_{2}\leq 0,\;0.01H\leq \mathrm{Rc}\leq 0.2H,\;4\mathrm{Rc}\leq \mathrm{Sc}\leq 8\mathrm{Rc}$. In total, a 750-simulation data set is used, while random data division is used to assign 70% as training, 15% as validation and 15% as testing set.

**Figure 11:**
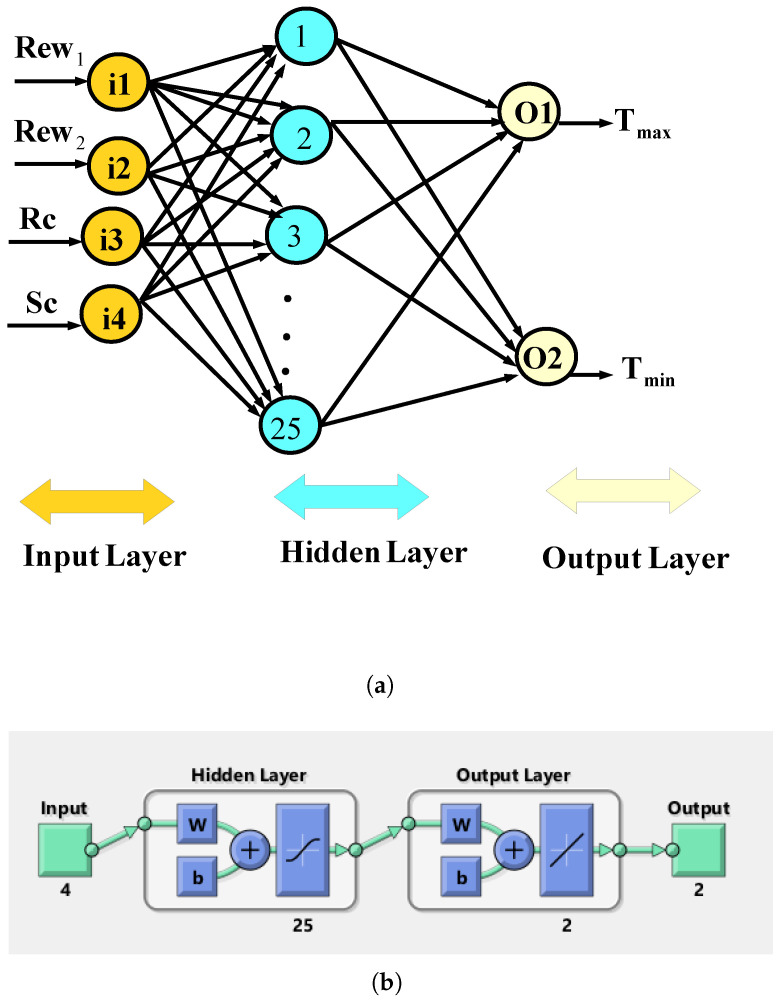
Schematic of the ANN with inputs–outputs (**a**) and network architecture with different layers (**b**).

In the ANN model, the output for the neuron model is written as:

(14) $$G_{j}=\sum_{i=1}^{N}W_{ij}R_{i}$$

where $R_{i}$ and $W_{ij}$ denote the ANN inputs and ANN weights. Different layers such as input, hidden and output layers are used, while in Figure 11a a schematic view is given. In the model, ANN weights are considered for the connection of layers, while every unit sums its inputs and bias terms. Figure 11b shows the ANN model structure with 25 neurons in the hidden layer.

**Figure 12:**
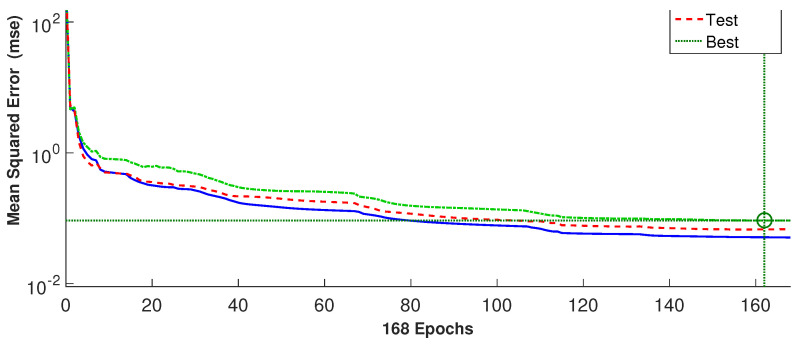
Variation of MSE for different epochs considering different data sets.

The number of the neurons is decided according to the performance criteria, which are the MSE (mean square error) and $R^{2}$ (coefficient of determination). They are given as:

(15) $$\mathrm{MSE}=\frac{1}{N}\sum_{i=1}^{N}{\left(y_{i}-y_{i}^{*}\right)}^{2},$$

(16) $$R^{2}=1-\frac{\sum_{i=1}^{M}{\left(y_{i}-y^{*}\right)}^{2}}{\sum_{i=1}^{M}{\left(y_{i}-{\bar{y}}^{*}\right)}^{2}},$$

where *N* and $\bar{y}$ denote the number of data and average values. As the learning technique, the Levenberg–Marquardt method with back-propagation is considered. In Figure 12, the performance of the ANN for different epochs is given. The MSE becomes lower as the iteration is progressed, while the weights are updated during the process. The best performance is achieved at epoch 162.

**Figure 13:**
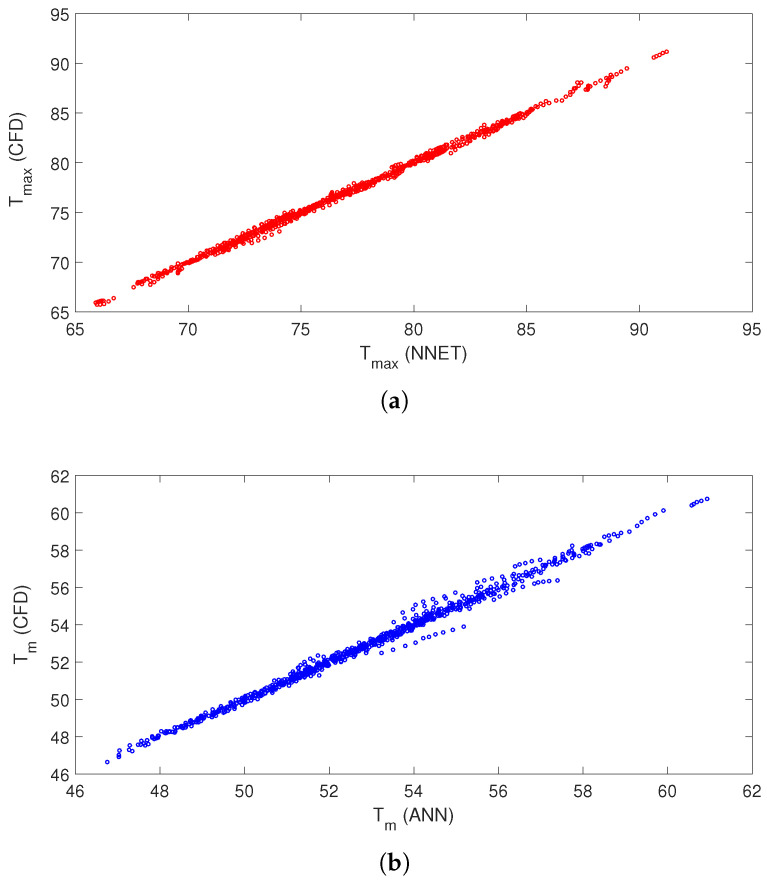
Estimation of maximum (**a**) and average (**b**) panel temperatures by using ANN.

Table 2 shows the ANN performance with 25 neurons in the hidden layer for the training, validation and testing data sets. The MSE values are lower while correlation coefficients are closer to 1 for all data sets. Estimated cooling performances in terms of maximum and average PST are shown in Figure 13a,b. The overall agreement of the ANN outputs and CFD outputs are satisfactory. The results reveal that ANN can predict the cooling performance of conducive panel when double RCCs are used.

## 4. Conclusions

Using double RCCs with multiple-slot nanojet impingement is considered as the cooling system for a conductive panel. Different rotational speeds and su-cooled temperatures are used for the RCCs. The following conclusions can be stated:

- When rotations of both cylinders become active, the average Nu rises while PST drops. As compared with motionless cylinders, 13% and 30.8% rises in average Nu are seen at RCC rotations of (−20,0) and (−20,−20), while PSTs are obtained as 2.14 ${}^{{}^{\circ}}$C and 5.84 ${}^{{}^{\circ}}$C.
- The distance and size of the D-RCCs are also influential on the cooling performance. The average PST is obtained as 7 ${}^{{}^{\circ}}$C with varying size, while it is only 1.75 ${}^{{}^{\circ}}$C with varying the distance between the RCCs.
- Subcooling of the active cylinders improves the cooling performances. The average Nu rises become 5.8% and 6% at the subcooled temperature of 10 for stationary and RCC cases, while PSTs are evaluated as 1 ${}^{{}^{\circ}}$C and 1.25 ${}^{{}^{\circ}}$C.
- When NFs are used, PST becomes 10 ${}^{{}^{\circ}}$C at the highest NP loading for the rotating case of RCC at the highest speed.
- The ANN-based model well predicts the maximum and average PST considering the parameters of the RCCs as the inputs and by using the high-fidelity CFD data.

## Figures and Tables

**Table 1:** Thermophysical properties of water and alumina at 25 ${}^{{}^{\circ}}$C [48].

Property	Water	Al${}_{2}$O${}_{3}$
Density (kg/m${}^{3}$)	997	3970
Specific heat (J/kg K)	4179	765
Viscosity (mPa.s)	0.895	-
Thermal conductivity (W/mK)	0.613	40

**Table 2:** ANN performance (variation of MSE and $R^{2}$) for different data sets.

Data Sets	Samples	MSE	R${}^{2}$
Training	524	$5.11\times 10^{-2}$	0.9998
Validation	113	$9.24\times 10^{-2}$	0.9997
Testing	113	$6.74\times 10^{-2}$	0.9998

## Data Availability

Not applicable.

## Abbreviations

The following abbreviations are used in this manuscript:

*A*${}_{1}$, *A*${}_{2}$	coefficient for viscosity
*C*${}_{k}$	coefficient for thermal conductivity
*D*${}_{h}$	hydraulic diameter, (m)
*h*	heat transfer coefficient, (W/(m${}^{2}$K))
*k*	thermal conductivity, (W/(m K))
KR	conductivity ratio
*H*	slot-to-plate distance, (m)
Nu	Nusselt number
*p*	pressure, (Pa)
Pr	Prandtl number
*R*	residual
Rc	cylinder radius, (m)
Re	Reynolds number
Rew	rotational Reynolds number
Sc	distance between the cylinders, (m)
*T*	temperature, (K)
*u, v*	velocity components, (m/s)
*w*	slot width, (m)
*W*	weight function
*x, y*	Cartesian coordinates, (m)
**Greek Characters**	
α	thermal diffusivity, (m${}^{2}$/s)
ϕ	solid volume fraction
ν	kinematic viscosity, (m${}^{2}$/s)
ρ	density, (kg/m${}^{3}$)
ω	rotational speed, (rad/s)
ΔT	temperature difference, (K)
**Subscripts**	
*c*	cold
*h*	hot
*m*	average
*nf*	nanofluid
*p*	solid particle
**Abbreviations**	
ANN	artificial neural network
EBE	energy balance equation
MSE	mean square error
NF	nanofluid
NP	nanoparticle
PST	panel surface temperature
PV	photovoltaic
PV-T	photovoltaic thermal
RCC	rotating circular cylinder
SVF	solid volume fraction
T-RCC	twin rotating circular cylinder
